# Supplementary material for: Are carbon emissions trading and green financial instruments synergistic? -Comprehensive quantitative research based on content analysis
Source: PLoS One. 2024 Mar 7;19(3):e0298601. doi: 10.1371/journal.pone.0298601 (PMC10919601; doi:10.1371/journal.pone.0298601)
Supplement: S1 Data — (ZIP) [file pone.0298601.s001.zip › Data/地级市Z_F工作报告.zip!/╔╜╬≈/╦╖╓▌╩╨/└┤╫╘╣1⁄2╓┌║┼í╛┬φ┐╦╩2╛▌═°í┐_2018.docx]

各位代表：

　　现在，我代表市人民政府向大会报告工作，请予审议，并请市政协委员和其他列席人员提出意见。

　　一、2017 年工作回顾

　　2017年，是我市发展进程中极不平凡的一年，也是我市经济持续稳中向好、奋力开创新局的一年。一年来，我们坚持以习近平新时代中国特色社会主义思想为指导，全面贯彻党的十九大精神和习总书记视察山西重要讲话精神，在省委、省政府和市委的坚强领导下，按照“一个指引、两手硬”的思路和要求，紧扣“两大任务”“五条新路”总体部署，团结带领全市人民，攻坚克难、开拓进取，全市经济社会发展各项工作取得新成就。

　　（一）经济运行稳中向好。主要指标增速进一步加快，多项位居全省前列。全市地区生产总值完成980.2亿元，增长7.3%，增幅排名全省第三；规上工业增加值完成289.5亿元，增长6.3%；公共财政收入完成73.2亿元，增长49.1%，增幅排名全省第二；固定资产投资完成214.2亿元，增幅由负转正，增长3.1%；社会消费品零售总额完成311.7亿元，增长7.1%；城镇居民人均可支配收入30989元，增长6.9%，增幅排名全省第二；农村居民人均可支配收入12305元，增长7.2%。

　　（二）产业转型成效明显。巩固提升煤炭、火电两大传统产业，培育壮大高端陶瓷、新能源新材料、现代煤化工、生物医药、文化旅游、草牧业和农产品加工、商贸物流等七大产业。实施产业转型项目133个，总投资573亿元。非煤产业投资占工业投资的81%。非煤工业增加值增长8.6%，高出规上工业增加值2.3个百分点。建成标准化矿井40座，其中现代化矿井6座。新增光伏、风电装机37.1万千瓦，新能源电力装机占比达36.7%。积极推动晋北（朔州）现代煤化工基地建设，松蓝化工2×40万吨丙烯酸项目前期工作有序推进。中美新能源闪氢提油中试顺利。成功举办第五届亚洲粉煤灰国际交流大会。国家大宗工业固废产品质量检验中心挂牌运行。工业固废综合利用产值达120多亿元，综合利用率达65%。旅游业综合收入实现203.9亿元，增长26.9%。

　　（三）改革开放不断深化。煤炭供给侧结构性改革取得实效，关闭煤矿4座，退出落后产能1360万吨。开发区改革创新发展扎实推进，朔州经济开发区、右玉生态文化旅游开发区、怀仁开发区挂牌运行，平鲁区、山阴县、应县设立开发区进展顺利；朔州经济开发区“三制”改革已完成，“三化”改革全面推开。旅游体制机制改革有序推进，市县（区）全部撤局设委，全市11个景点景区完成管理权和经营权分离。国企国资改革完成年度任务。医疗卫生体制改革取得新进展，9所城市公立医院取消药品加成，六县（区）全部组建医疗集团，县乡医疗卫生机构一体化改革实现全覆盖。电力体制改革有了新突破，交易直供电量21.4亿度，培育了6家售电公司，山阴北周庄工业园区列入全国增量配电业务改革试点。实施12个PPP项目，其中7个完成招标，3个开工建设。朔州农村商业银行筹建工作有序推进。晋坤公司“新三板”挂牌上市。全市新增高新技术企业10家，总数达到21家，省级重点实验室和工程技术中心达到2家。招商引资取得新成果，全年签约项目205个，总投资1126.8亿元。

　　（四）“三农”工作扎实推进。农业供给侧结构性改革取得新进展，饲草饲料作物种植面积达到177万亩，比上年增加17万亩。实施规模健康养殖，改造提升58个养殖小区，奶牛存栏19万头，肉羊饲养608万只。承办了全国杂粮绿色高产高效模式交流会，举办了全国北方农牧交错带农业结构调整现场会。农业产业化经营组织达到1218家，农产品销售收入达到205.5亿元，增长7%。累计认定“三品一标”农产品生产基地133万亩，认证“三品”农产品130个，地理标志认证产品8个。开展了“三环三边”“四治四无”集中整治行动，实施农村危房改造2485户，农村人居环境得到进一步改善。神头镇等5个村镇荣获第五届全国文明村镇称号，山阴县燕庄村被评为全国改善农村人居环境示范村。农村改革不断深化，农村承包地确权登记颁证基本完成。启动农村集体产权制度改革，市、县两级农村产权流转交易市场全部建成。

　　（五）城市功能更趋完善。新开工保障性住房6903套，基本建成8725套，完成投资17.69亿元。公租房分配圆满完成，全市4044户低收入困难居民喜迁新居。新建改建城市道路60.6公里、市政管网344.5公里，新增供热面积700万平方米。七里河综合治理初见成效，敬德公园正式开放。“三路五桥”建设顺利推进，市区振华东街改造完成。数字化城管平台基本建成。实施民福街西延线、市府街西延线等七条道路绿化改造，国家园林城市创建成果进一步巩固。市区新增纯电动公交车100台，新增公交运营线路26公里，延伸农村线路15公里，覆盖38个周边村镇。

　　（六）生态环境明显改善。完成营造林12.3万亩，治理水土流失面积29.7万亩，桑干河综合治理与生态修复五大水利项目全部启动。实施冬季清洁取暖改造工程，完成5.7万户改造任务。淘汰燃煤小锅炉76台，取缔“散乱污”企业443家。全年空气质量综合指数5.9，全省排名第二，下降8.7%，下降率全省排名第一。细颗粒物（PM2.5）平均浓度每立方米48微克，全省排名第二，下降15.8%，下降率全省排名第一。优良天数242天，全省排名第三。推进城市污水处理设施建设。实施了矿井外排水质治理提标改造工程。市、县、乡、村四级全面落实河长制。完成地下水关井压采任务，省考核的5个地表水断面水质全部达标，万元工业增加值用水量较上年降低2.2%，万元GDP用水量较上年降低6.1%。中央和省环保督察组交办的395件问题事项全部办结，反馈的34件问题事项整改已达序时进度。

　　（七）民生事业全面进步。坚持精准扶贫、精准脱贫，预计国家级贫困县右玉县和省级贫困县平鲁区、山阴县整体摘帽，24095人脱贫，148个贫困村退出，实现脱贫攻坚再战再胜。全市提前一年全部通过县域国家义务教育均衡发展验收。高中教育质量明显提升，全市二本以上达线人数14111名，创历史最好水平。朔州大医院项目顺利推进。城镇新增就业等6项指标全部达标。机关事业单位养老保险制度实现入轨运行。城乡居民基本医疗保险完成整合，与省内10家定点医药机构开通了异地就医直接结算。参加省十五届运动会资格赛，获得五金八银十三铜的好成绩。公共文化服务体系进一步完善。狠抓安全生产工作，全年发生生产安全死亡事故19起，同比减少6起，下降24%。社会治安综合治理进一步加强，防控体系进一步完善，有力维护了社会和谐稳定。

　　（八）政府建设持续加强。深化“放管服效”改革，取消行政职权、中介服务事项59项。强化政务服务，实行“一个窗口受理、一条龙服务、一站式办结”，全年办结业务19.4万件，办结率99.98%。严格执行“13710”督办制度，有效促进了工作落实。自觉接受人大、政协监督，支持各民主党派、工商联、无党派人士参政议政，办理人大代表建议124件、政协委员提案112件。

　　国防后备力量建设进一步加强，双拥工作扎实开展。妇女、儿童、老龄、残疾人、信访、宗教、人防、地震、应急、气象、档案、地方志、红十字会等各项事业都取得新成绩。

　　各位代表，过去一年取得的成绩来之不易。这是全市上下深入贯彻习近平新时代中国特色社会主义思想和党的十九大精神的结果，是省委、省政府和市委正确领导的结果，是市人大、市政协有效监督、全力支持的结果，是全市人民齐心协力、共同奋斗的结果。在这里，我代表市人民政府，向全市人民和所有关心支持朔州发展的各界人士，表示衷心的感谢和崇高的敬意!

　　在肯定成绩的同时，我们也要清醒地看到，我市发展不平衡不充分的问题比较突出，仍面临不少困难和挑战。集中表现为：新兴产业发展缓慢，转型发展任重道远；大项目好项目储备不多，投资增长乏力；科技创新和人才支撑不够，发展动力不足；对外开放水平不高，招商引资难度越来越大；基础设施建设滞后，城市的吸引力、辐射力、带动力不强；民生改善任务繁重，生态环境治理压力较大；安全生产形势不容乐观，社会治理还有不少薄弱环节；政府职能转变还不到位，一些干部仍存在“无所谓、不作为、不担当、落实差、花架子”的问题，作风建设须进一步加强。我们一定要安不忘危、兴不忘忧，切实强化底线思维、增强忧患意识，下大力气补短板、强弱项、解难题，尽心竭力做好政府工作！

　　二、2018 年工作目标

　　2018年是贯彻落实党的十九大精神的开局之年，是改革开放40周年，是决胜全面小康、实施“十三五”规划承上启下的关键一年。我们要高举习近平新时代中国特色社会主义思想伟大旗帜，全面深入贯彻党的十九大和十九届二中、三中全会精神，按照省委省政府“一个指引、两手硬”的思路要求和“三大目标”定位，在市委的坚强领导下，坚持稳中求进工作总基调，坚持新发展理念，坚持高质量发展，主动适应社会主要矛盾变化，紧扣“两大任务”工作主题和“五条新路”主攻方向，大力学习和弘扬右玉精神，始终把深化供改与深化综改相结合作为经济工作主线，统筹推进稳增长、促改革、调结构、惠民生、防风险各项工作，更加注重生态优先、绿色发展，更加注重质量第一、稳煤促新，更加注重项目攻坚、人才支撑，更加注重改革开放、创新驱动，更加注重民生至上、创安维稳，大力推动质量变革、效率变革、动力变革，坚决打好防范化解重大风险、精准脱贫、污染防治攻坚战，找准争当能源革命排头兵新站位，建设美丽宜居新家园，打造践行“两山”理论示范区，形成对内对外开放新格局，创造人民群众获得感幸福感安全感持续增强的美好生活，奋力朝着全面建成小康社会的宏伟目标迈进。

　　主要预期目标是：地区生产总值增长7%左右，规模以上工业增加值增长6%左右，全社会固定资产投资增长15%，公共财政预算收入增长7.5%，社会消费品零售总额增长7%左右，城乡居民人均可支配收入分别增长7%和7%以上，城镇新增就业2.3万人，城镇调查失业率、城镇登记失业率分别控制在6%、4.2%以内。其他约束性指标完成省下达任务。

　　完成全年目标任务，我们要坚持和把握“六个必须”。

　　一是必须认真落实习总书记重要批示指示精神，把右玉精神作为推动发展的力量源泉。右玉精神是朔州宝贵的政治资源和精神财富。我们要深入学习贯彻习总书记对右玉精神的重要批示指示精神，认真落实省委、市委关于学习弘扬右玉精神的部署要求，持续深化“右玉精神在朔州”活动，在践行中传承，在传承中弘扬，使右玉精神成为提升境界、凝聚力量、推动发展的强大动力。

　　二是必须突出高质量发展，把项目建设作为推动转型的重要抓手。高质量发展是我们经济工作追求的目标，转型发展是我们必须要过的坎，早转早好。项目是转型的“硬支撑”，没有项目带动，转型发展只能是空谈。要牢固树立抓项目就是抓发展的理念，始终把项目建设作为转型发展、高质量发展的关键抓手，紧盯不放、锲而不舍，步步为营、久久为功，为转型发展提供强大支撑。

　　三是必须坚持供改综改相结合，把改革创新作为引领发展的关键一招。我们要向改革要活力，向创新要动力。坚持大胆实践、勇于改革，先行先试、敢于创新，以改革促转型。协同推进全方位改革、全领域创新，不断破除发展道路上的“拦路虎”和“绊脚石”，使体制机制优势转化为发展优势，使政策制度创新转化为发展红利。

　　四是必须解放思想敞开胸怀，把扩大开放作为开拓发展空间的必由之路。我们的城市因开放而建，对外开放是这座城市的优秀基因，更是我们需要大力弘扬的时代精神。我们不仅要在已有的传统领域“提质”“输出”，而且要进一步解放思想，以“无中生有”的思维来“创意”“引入”，以“只争朝夕”的劲头来谋招商、抓落地，推动朔州尽快融入环渤海、对接京津冀、拉手雄安新区，再造发展新优势。

　　五是必须做大做强县域经济，把城乡区域协调发展作为实现全面小康的有力支撑。县域经济是朔州经济的“脊梁”。我们的县域经济优势和劣势并存，优势明显，潜力巨大，独立性和能动性很强。我们要增强信心、超前谋划，抓住国家实施乡村振兴战略的重大政策利好，把握交通区位优势快速提升和产业梯度转移带来的重大机遇，优化产业布局。要进一步树立强烈的市场意识、竞争意识、进取意识，瞄准产业发展方向，把特色优势干成区域乃至全国的“单项冠军”。同时要强化县域之间互联互通，形成竞相发展、差异发展、抱团发展的生动局面。

　　六是必须强化招才引智，把人才强市作为提升竞争力的优先战略。转型发展靠人才。人才是我们的致命短板，要舍得投入。我们一定要牢固树立“人才是第一资源”的理念，紧紧抓住新一轮人才配置机遇，主动积极作为,制定更加优惠的政策，留住本土人才，用好外来人才，种好“梧桐树”，引得“凤凰”来。

　　三、2018 年主要任务

　　按照省委、省政府安排部署，结合我市实际，今年要着重做好八方面工作。

　　（一）重点打造“2＋7”产业格局

　　大力推动产业优化升级。围绕“2＋7”产业布局，推进一批引领带动产业转型的大项目、好项目，扶持一批具有良好支撑性、引领性、成长性的优势企业。煤炭产业方面，按照“减、优、绿”的要求，抓好减量置换和减量重组，持续提升先进产能比重，安全高效生产煤。加大技改力度，推动标准化矿井建设，全市标准化矿井保持在40座以上。抓好东露天界内地方煤矿整合、平鲁区芦家窑煤业配采等项目建设。火电产业方面，加大燃煤机组超低排放改造力度，淘汰落后产能，提升电力发展质量。充分利用蒙西至天津南、晋北至南京两条特高压输电线路，大力开拓市场，扩大外送电量。抓好神电“上大压小”2×100万千瓦和华昱二期2×35万千瓦、同煤朔南2×35万千瓦、平朔木瓜界2×66万千瓦等3个低热值煤发电项目建设。陶瓷产业方面，落实《朔州市促进陶瓷产业做大做强若干措施》等政策，引进建筑瓷、日用瓷、工业瓷项目，做大产业规模。支持企业技术创新，提高产品的技术含量和附加值。加大营销力度，打造朔州知名陶瓷品牌，建设中国日用瓷生产基地。4月份召开山西朔州陶瓷产品进出口交易会，邀请100多家外商参加展会；做好中国（广州）进出口商品交易会参展工作，开辟新的日用瓷出口通道。支持成立陶瓷行业联合会。强化产业发展基础支撑，建成山西省日用陶瓷产品检测检验中心，推进怀仁陶瓷职业技术学院建设。抓好华伟建陶高档内墙砖和鸿达、盛泽瓷业日用瓷生产线等项目建设。新能源新材料产业方面，大力发展风电、光伏发电、生物质能发电，提高清洁能源比重，建设国家级光伏发电应用领跑基地、晋北风电基地。抓好西易能源9.8万千瓦风电、茂华平鲁10万千瓦风电、晋能清洁能源2万千瓦光伏发电等项目。积极推进国家级区域工业绿色转型发展试点城市、全国工业固废综合利用示范基地建设，加大煤矸石、粉煤灰、脱硫石膏综合利用力度，推动“一园多企”提质增效，打造工业绿色产业走廊。抓好山西超牌20万吨煅烧高岭土、怀仁理思新型节能生产线二期、山阴玉竹新材料2万立方硅酸钙保温板等项目建设。现代煤化工产业方面，加快建设晋北（朔州）现代煤化工基地，尽快完成基地规划环评,与总体规划一并上报国家相关部委。抓好松蓝化工2×30万吨煤制乙二醇等项目建设。生物医药产业方面，引进国内外知名药企，建设新项目，开发新产品，扩大产业规模。抓好怀仁医药产业园、应县（北京）康庭阳光黄芪提取产业化、吉呈生物二期扩建工程等项目建设。文化旅游产业方面，坚持“举右玉龙头、走生态之路、打长城品牌”，围绕建设“塞上绿洲、美丽朔州”，精心打造内外长城、雁门边塞、应县木塔、西口古道等旅游品牌，挖掘布局农牧交错带、民族融合地、桑干河神头泉、金沙滩杨家将等特色资源，加大宣传推介力度，培育文化旅游产业新的增长点。支持右玉县国家全域旅游示范区和省级生态文化旅游开发区建设。实施古长城旅游公路、长城文明守望、长城生态绿化、长城沿线乡村环境整治、长城旅游基础设施建设“五大工程”。开工建设110公里古长城旅游公路。引进战略投资，加强与省文旅集团合作。抓好右玉杀虎口文化旅游区一期、神头镇湿地公园、南邢家河风情小镇、怀仁清凉山滑雪场等重点项目建设。5月份举办首届塞上（朔州）长城国际旅游节。草牧业与农产品加工业方面，把草牧业打造成农业经济的支柱产业、农民增收的主要渠道，把农产品加工作为龙头，构建一二三产融合发展的特色现代农业产业体系。抓好平鲁羔羊肉深加工、中藜食品藜麦加工、大象集团生猪养殖等项目建设。商贸物流业方面，强力推进平鲁内陆港建设，抓好怀仁海宁皮革城等重点物流项目，加快建设区域性特色物流中心。

　　建立项目推进机制。落实项目联审联批、领导包联、目标责任、督查考核、奖惩兑现等五项制度，推行企业投资项目承诺制试点，建立重大项目协调调度机制，积极解决手续办理、征地拆迁、环评、阻工等实际问题。按照“转型项目建设年”要求，开展“项目集中开工月”活动，4月份集中抓好开复工。

　　加快发展县域经济和民营经济。县区和开发区要放宽搞活，要看到我们财政负债额度小，仍有融资空间；要看到我们地广人稀有优势，土地改革有新机遇，进一步破解信贷、土地等瓶颈，解决项目落地的“痛点”。要全面优化环境，加大企业帮扶力度，激发民间投资。要健全企业家参与涉企政策制定机制。选树先进典型，大力弘扬企业家精神，全年力争培育小微企业1600户、小升规企业15户，培训企业家1000名，为活跃全市经济培育一批心怀高远但不好高骛远、勇于冒险但不铤而走险、洞察商机但不钻营投机、思想独立但不骄横自大、以企为家但也兼顾小家的优秀企业家。

　　增强财政金融服务实体经济能力。市财政专门预算转型发展资金5000万元，用于企业融资担保、产业发展基金、项目贷款贴息等，对七大产业重大支撑性招商引资项目，给予投资性奖励或基础设施配套。积极与国家、省内各大产业投资基金合作，组建配套产业发展基金，构建企业主导，政府、投资机构、民间资本和银行多元投入机制。壮大市、县政策性担保机构实力，降低担保成本，满足中小微企业融资担保需求。完成组建朔州农村商业银行，推进农村信用社改制。破解中小微企业资产不确权、产权不清晰不完整等融资瓶颈，引导各类企业进行股份制改造。支持行业内优势企业实施股权融资。推动玉龙化工“新三板”上市。始终做到财税金融底子清楚，着力防范化解财政金融风险。

　　（二）进一步深化改革开放

　　深化开发区改革创新。将开发区作为转型综改的主要突破口，优先在开发区布局产业转型项目。积极复制推广先行先试经验，全力推进“三化三制”改革。按照“能放尽放、能授尽授”原则，完成市、县两级政府对所有开发区的授权，实现“办事不出区”。支持开发区基础设施、公共服务平台、产业转型升级、重大投资项目建设，鼓励引导社会资本参与开发区基础设施和公共服务平台建设。

　　推动国企国资改革。加快剥离国有企业办社会职能，全面完成国有企业“三供一业”分离移交，完成医疗、教育机构重组、撤并和剥离，完成市政、社区剥离以及消防机构分类改革。进一步完善国有企业公司制改革，理顺国有资产监管体制。

　　推进电力体制改革。有序扩大市场化交易电量规模，积极发展售电公司。加快晋电外送电源点接入进度，推进江苏国信集团苏晋公司落户朔州。推动建设国家增量配电业务试点和固废综合利用厂用电试点。

　　提升对外开放交通能力。抓好“铁公机”建设，深化朔州机场前期工作，加速大原高铁朔州段开工，推动朔神高速复工，建成右平高速。启动县区之间快速路连接线前期工作。

　　高效精准招商引资。充分发挥各地朔州商会及中煤、阳煤、同煤等大集团作用，吸引境内外客商投资合作。充分利用蒙西至天津南、晋北至南京两条电力通道，吸引京津冀、长三角企业来朔投资。开发区要充分发挥产业转型主战场作用，围绕主导产业，提高项目策划包装水平，谋划一批重大产业转型项目。针对重点客商和重点项目量身打造个性化招商方案，实行“一企一策”，开展一对一专人负责，点对点对接洽谈，零距离跟踪服务，第一时间协调解决问题，有效促进项目落地投产。

　　扩大出口规模。巩固扩大出口市场份额，寻求新的增长点，提高经济外向度。重点扶持右玉图远脱水蔬菜，平鲁晋坤矿产品，怀仁晋利糖果、尊屹陶瓷、诺成制药，应县博达瓷业等企业拓展出口市场。

　　（三）深入实施创新驱动

　　加强科技创新。市政府安排1000万元专项资金用于科技创新，企业也要加大研发投入。推动中煤平朔公司技术中心申请认定国家级企业技术中心，山西雅士利、应县优尊陶瓷技术中心申请认定省级企业技术中心。扶持晋坤公司、朔神新材料等5家企业申报省级重点实验室、工程技术研究中心。鼓励支持有条件的企业申报国家或省科技重大专项项目。支持中美新能源粉煤加氢快速热解技术、北大研发中心粉煤灰制备高强纤维、三元炭素内串石墨化等重大科研成果工业化应用。依托朔州科技创新创业园，加强创新平台建设。支持中小企业创新发展，建设科技创新风险投资基金和中小微企业双创基地。

　　强化人才支撑。落实我市深化人才发展《意见》。设立人才发展专项资金，支持人才的培养、引进、开发、使用、奖励等。积极创造条件为人才创业提供投资担保，对引进人才实行生活补贴。支持三元炭素、晋坤矿产品、中美新能源等企业建立院士、博士工作站。聘请专业咨询团队，服务朔州建设发展。鼓励朔州企业走出去，与全国知名高等院校、科研院所深度合作，培育一批本土管理和实用技术人才。打好“乡音、乡缘、乡情”牌，鼓励吸引朔州籍科学家、企业家、艺术家关注家乡、服务家乡、建设家乡。发挥山西生态农牧研究院作用，为农牧业发展提供技术支持。对高端人才引进，实行“一事一议”，坚决兑现有关政策。

　　（四）大力推进乡村振兴

　　科学制定发展规划。按照“产业兴旺、生态宜居、乡风文明、治理有效、生活富裕”的总要求，科学规划主导产业、乡村风貌、文化特色、基础设施建设、基本公共服务、支撑政策体系。制定乡村振兴发展规划，市县两级6月底完成总体规划，9月底完成专项规划。

　　发展特色现代农业。坚持草畜一体、种养结合、农牧循环，建设全国草牧业示范市和雁门关农牧交错带核心样板区。突出抓好180万亩饲草料、100万亩优质杂粮、100万亩经济作物和40万亩马铃薯基地建设，推进32个有机旱作农业示范园建设。实施品种改良，推动基础母羊建设，打造怀仁牧繁农育和右玉等山坡区自繁自育800万只肉羊产业区，打造山阴、应县20万头现代奶牛产业区。9月份举办首届怀仁羔羊肉国际交流大会。

　　提升农业社会化和产业化水平。围绕草食畜、杂粮、蔬菜、油料、马铃薯、中药材、饲草料、林果等产业，加大“三品一标”认证和品牌创建。培育新型农业经营主体，发展多种形式的适度规模经营，扶持一批农产品加工龙头企业。拓宽农产品销售渠道，促进农村电商发展，力争全年农产品加工销售收入达到218.5亿元，增长6.5%以上。培育发展农业社会化服务组织，建设畜产品质量检测检疫中心。加大补贴力度，不断完善农业巨灾风险和市场风险的分散机制。市财政安排906万元，专项用于农业保险补贴。

　　深化农村改革。全面完成农村承包地确权登记颁证。开展农村集体三资清产核资工作。发挥省级试点应县和朔城区南邢家河村的示范作用，整体推动全市农村集体产权制度改革。市县乡三级农村产权流转交易中心全部运行。抓紧启动农村闲置宅基地和闲置农房整治盘活利用工作。

　　推进美丽宜居乡村建设。实施农村人居环境改善“四大工程”，加强农村基础设施建设。抓好总投资5.14亿元的农村生活垃圾清理和污水治理项目。开工建设450公里“四好农村路”。规划50个长城文化、农牧主题的美丽乡村，建成12个休闲农业和乡村旅游示范点。鼓励各县（区）推进田园综合体建设，支持右玉打造全省试点县。探索发展富有晋北乡村特色的民宿和康养基地。5—10月份举办首届休闲农业暨乡村旅游季活动。

　　强力推进脱贫攻坚。按照摘帽不摘责任、不摘政策、不摘帮扶、不摘监管的要求，继续加大帮扶力度，加大资金投入，全面巩固脱贫成果，圆满完成剩余4654名贫困人口减贫任务。

　　（五）不断提升城镇化水平

　　加快市城区建设。加大规划编制力度，推动“多规合一”。开工迎宾大道、奥体中心、垃圾填埋场、城中村改造等46个新建项目，加快推进朔州大医院建设，完成七里河五座桥梁、恢河大桥、地下管廊、污水处理厂改造提升、7条小街小巷改造提升、42座公厕新建改建等基础设施建设。对人民公园、市府广场等11个公园，市府街、民福街等8条道路进行绿化提质。启动公交调度中心、候车厅和充电桩建设改造工程。推进智慧城市建设。深化城市综合执法体制改革，创新城市管理方式，提升城市精细化管理水平。完善城市应急体系，保障城市安全运行。

　　提升县城建设水平。实施设施提升、城市安居、城中村改造、环境提质“四大工程”。引导生产要素、优势资源向县城集聚，促进人口向县城集中，建设和谐宜居、富有活力、各具特色的大县城。

　　推进特色小镇建设。抓好右卫镇、金沙滩镇2个全国特色小镇建设，推动北周庄镇、凤凰城镇、威远镇、南河种镇申报第三批全国特色小镇。提高城镇人口承载能力，培育更多示范集镇，打造一批经济强镇、商贸重镇、旅游名镇。

　　深入推进“六城联创”。巩固国家园林城市、全国双拥模范城创建成果，加快国家卫生城市、国家环保模范城市、国家文明城市、国家食品安全城市创建步伐。

　　（六）持续推进生态文明建设

　　坚持植绿兴水并重。统筹山水林田湖草系统治理，开展更大规模植树造林，力争到2030年实现全市宜林荒山全部绿化，怀仁、右玉2020年率先完成。今年完成营造林33.48万亩，重点抓好“一城两线”植树造林，推进朔城区南山生态综合治理二期4万亩、右玉县通道两侧荒山造林4万亩等18项工程，掀起全民动员、绿化家园的热潮。加大经济林种植力度，力争新建10个千亩以上经济林示范园，种植经济林7万亩以上。坚持“浚河、控污、固堤、绿岸、增水、兴业”，扎实推进“一山两河一湖”生态建设，抓好桑干河综合治理和生态修复，启动永定河生态修复综合治理项目。继续实施引黄永定河生态补水，推进全市水资源全域化配置和神头泉水优水优用。投资1.2亿元，解决平鲁、山阴、右玉部分山区175个村、7.18万人、57万牲畜安全饮水问题。

　　打好大气、水和土壤污染防治“三大战役”。深入开展“铁腕治污”行动，切实形成严管重处机制，做实“二污”普查和生态红线划定工作，坚决完成中央、省环保督察组反馈问题事项整改，推进治污攻坚任务落实。大气污染防治方面，深化“控煤、治污、降尘、管车”措施，推动大气质量持续改善。大力推进煤改电、煤改气和热电联产集中供热工程，抓好总投资8.3亿元的12个集中供热项目，完成市城区100%、县（区）建成区50%以上、农村30%以上燃煤户清洁取暖改造任务。推进昱光、朔南、木瓜界热电联供电厂管网建设。持续开展“散乱污”企业排查整治，坚决杜绝“散乱污”企业死灰复燃。坚持禁燃禁放常态化，消除煤矸石自燃，严格控制秸秆焚烧。专项治理散煤短途运输，降低道路扬尘污染。水污染防治方面，启动城乡垃圾污水治理50个项目。强化城镇生活污水治理，完成城镇污水处理厂提标改造和扩建工程，完善城镇污水管网接入工程。加快工业集聚区污水集中处理设施建设，积极开展化工、制药、煤炭、陶瓷、制革等行业清洁化改造。强化农业农村污染治理，完成省定农村环境综合整治任务。抓好城镇和农村集中式饮用水源地保护区划分、报批、规范化建设，推进污染源取缔工作。实施最严格的水资源管理制度，深入推进河长制，加大桑干河流域污染治理力度。土壤污染防治方面，加强固体废物特别是煤矸石、粉煤灰综合利用和规范化处置，严格危险废物监管，防范土壤环境风险。

　　强化生态治理修复。实施采煤沉陷区、地质灾害、裸露山体治理，推进矿山生态环境治理。加快推进4个县区15个乡镇55个村1.66万户4.54万人的采煤沉陷区治理搬迁安置。完成500户农村地质灾害治理搬迁任务。

　　（七）切实保障和改善民生

　　优先发展教育。巩固提升义务教育均衡发展成果，推动“局管校聘”试点工作，强化师德师风建设。以均衡编班、均衡配备教师为突破口，保障特殊群体就学需求，解决“择校热”“大班额”问题。开工建设市一中新校区等10所学校，建成20所普惠制幼儿园。实施高中阶段教育普及攻坚计划，积极推进普通高中学校标准化建设。加快推进教育信息化建设。

　　提升卫生健康服务水平。完成县乡医疗卫生机构一体化改革任务。建设全员人口健康信息平台。全面推开医联体建设，不断完善分级诊疗体系。提升乡镇卫生院和社区卫生机构服务能力，为97所乡镇卫生院分别安排保障性资金10万元。

　　扩大就业创业。培育创业创新公共平台，壮大创业担保贷款规模，加大减税降费力度，充分调动社会大众的创业积极性，推进创业带动就业。加强对困难人员的就业援助，推进农村劳动力转移就业。健全覆盖城乡的公共就业创业服务体系，做好城镇失业人员等群体的就业工作，托底帮扶就业困难人员。

　　完善社会保障体系。全面建立城镇职工、城乡居民社会统筹和个人账户相结合的社会养老保险制度。城乡居民基本医保人均财政补助标准再增加40元，一半用于大病保险。深化医保支付方式改革，扩大异地就医直接结算范围。建立健全医保打包付费制度。投资1500万元，新改扩建乡镇敬老院5所、农村老年人日间照料中心140个。市社会福利院老年公寓、市残疾人托养中心建成使用。实施五保供养机构公建民营改革，向社会提供更优质的养老服务。

　　推进住房保障工作。投资12亿元，新开工城镇棚户区改造4374套，基本建成3030套。为2300户城镇住房保障家庭发放租赁补贴。

　　提高城乡居民收入。拓宽城乡居民劳动收入和财产性收入渠道，特别是要提高农民收入水平。开展“农村本土人才回归工程”，鼓励引导农民群众返乡创业，培训1000名农村各类实用人才。壮大农村集体经济，村集体收入5万元以上的村达到60%。

　　推动文化体育事业发展。大力践行社会主义核心价值观，唱响主旋律，弘扬正能量。进一步完善公共文化服务体系，加大文化遗产保护力度，推进乡村文化记忆工程，繁荣文化和文艺创作，广泛开展群众文化活动和全民健身活动。实施文化惠民工程，送戏下乡1100场。深入挖掘整理朔州历史文化，增强全市人民的文化自信和文化认同，凝聚团结力量。推进右玉“全国足球夏训基地”22个足球场建设，支持右玉办好全域生态国际马拉松赛事。

　　（八）全力维护社会安全稳定

　　加强安全生产工作。压实党委政府安全生产工作领导责任、部门监管责任、企业主体责任，扎实开展安全生产“严格执法年”活动，严格执行隐患排查治理、重大隐患挂牌督办、追责问责、联合惩戒“四个清单”制度，严厉打击安全生产非法违法行为。全面落实安全生产领域改革发展和推进城市安全发展实施意见，健全完善安全考核评价制度。加大安全生产投入，加强应急救援队伍和应急储备物资建设。重点抓好煤矿、道路交通、建筑工地、人员密集场所、消防、危化品、防汛、森林防火等重点领域的安全生产工作，特别是要严厉打击私挖滥采、超层越界开采、关闭矿井死灰复燃，杜绝重特大事故发生，促进安全生产形势持续稳定好转。

　　加强和创新社会治理。坚持共建共治共享，不断提升社会治理社会化、法治化、智能化、专业化水平。健全公共安全体系，全面推进社会治安防控体系建设，进一步完善长效工作机制。实施质量强市战略，促进质量安全根本改善。加强食品药品安全监管。坚持“零容忍”态度，深入开展扫黑除恶专项斗争，严打黑恶势力犯罪。创新信访工作机制，有效化解各类矛盾纠纷，解决好群众合理合法利益诉求，依法打击缠访闹访、以访谋利等行为，维护社会和谐稳定。

　　同时，支持国防建设和军队改革，推进军民融合，强化基地训练，支持驻军停止有偿服务，聚焦备战打仗。支持工会、共青团、妇联等群团组织开展工作，发展妇女、儿童、老龄、慈善和红十字会等事业，加强气象、地震、人防、科普、经济普查、档案等工作，做好民族宗教、外事、侨务、对台等工作，为我市改革发展凝聚强大合力！

　　四、建设人民满意的服务型政府

　　新时代新使命对政府自身建设提出新要求。我们要始终坚持为民、务实、清廉的价值追求，进一步提高执政能力和执政水平，全力打造人民满意的服务型政府。

　　强化学习型政府建设。认真学习习近平新时代中国特色社会主义思想，扎实开展“不忘初心、牢记使命”主题教育，树牢“四个意识”，坚决维护以习近平同志为核心的党中央权威和集中统一领导。大力弘扬右玉精神，营造乐于学习、勤于调研、勇于实践的浓厚氛围，切实提升政府工作人员特别是领导干部的学习本领、政治领导本领、改革创新本领、科学发展本领、依法执政本领、群众工作本领、狠抓落实本领、驾驭风险本领等“八种本领”。

　　强化法治政府建设。突出抓好宪法学习宣传和贯彻实施，不断增强宪法意识，切实维护宪法尊严和权威。坚持依法行政，把政府各项工作纳入法治轨道，切实提高依法办事水平。建立重大行政决策合法性审查机制、终身责任追究制及责任倒查机制，推进决策科学化、民主化、法治化。发挥好政府法律顾问作用。严格执行人大及其常委会的决议决定，自觉接受人大、政协监督，认真办理代表建议和委员提案，听取民主党派、工商联、无党派人士和各人民团体意见，做到件件有落实，事事有回音。

　　强化效能政府建设。严格按照中央、省委统一部署，蹄疾步稳推进地方机构改革。深化“放管服效”改革，做好审批事项承接、取消、下放、调整工作。全面深化、拓展、延伸“13710”信息化督办制度。强化政务服务，推行“最多跑一次”改革。全面落实“1＋9”专项行动，全力营造审批最少、流程最优、体制最顺、机制最活、效率最高、服务最好的“六最”营商环境。

　　强化实干型政府建设。市委作出的决策部署，市政府就要狠抓落实。要大力开展“大学习、大调研、大落实”活动；深入开展“力戒形式主义，狠抓工作落实”作风整顿，强班子、带队伍、抓落实。要盯住咬紧，一抓到底，抓出成效。着力整治“无所谓、不作为、不担当、花架子、落实差”和“吃拿卡要”等不良风气。严肃问责不作为、慢作为、乱作为等行为。

　　强化廉洁政府建设。切实扛起全面从严治党主体责任，认真履行“一岗双责”。严格贯彻执行中央八项规定精神，持续狠刹“四风”，严控“三公”经费，让有限的财力优先保障民生。强化对行政权力的监督和制约，严格监管公共资金、公共资源、国有资产。扎实开展重点领域和关键环节专项整治，坚决纠正侵害群众利益不正之风。深入开展党风廉政建设和反腐败斗争，持续形成干部清正、政府清廉的良好局面。

　　各位代表，朔风迎春暖，时代启新篇。让我们更加紧密地团结在以习近平同志为核心的党中央周围，高举习近平新时代中国特色社会主义思想伟大旗帜，在省委、省政府和市委的坚强领导下，埋头苦干、攻坚克难，为决胜全面建成小康社会、谱写新时代中国特色社会主义朔州篇章而努力奋斗！
